# Smoking Habits and Workplace Health Promotion among University Students in Southern Italy: A Cross-Sectional Pilot Investigation

**DOI:** 10.3390/ijerph191710682

**Published:** 2022-08-27

**Authors:** Elpidio Maria Garzillo, Maria Grazia Lourdes Monaco, Anna Rita Corvino, Alessia Giardiello, Antonio Arnese, Francesco Napolitano, Gabriella Di Giuseppe, Monica Lamberti

**Affiliations:** 1 Department of Prevention, Abruzzo Local Health Unit No. 1, 67100 L’Aquila, Italy; 2 Occupational Medicine Unit, University Hospital of Verona, 37134 Verona, Italy; 3 Department of Experimental Medicine, University of Campania ‘Luigi Vanvitelli’, 80138 Naples, Italy

**Keywords:** workplace health promotion (WHP), tobacco smoking, medical students, health professional students, health promotion, occupational health, public health

## Abstract

The study aimed to investigate the tobacco smoking prevalence, habits and awareness among a cohort of healthcare students from a university hospital in southern Italy and the associations with socio-demographic determinants. A secondary outcome was to estimate the educational needs to receive information on smoking-related risk factors. Five hundred and forty-nine students completed a self-administered questionnaire (180 male and 369 female, average age 25 yo, ±5.9 SD), enrolled from October 2018 to November 2019 at the University of Naples ‘Luigi Vanvitelli’, and the collected data were analysed by descriptive and inferential statistical analysis. The sample’s prevalence of current smokers was 25.3%, without a significant sex difference. The multiple logistic regression model showed the link between smoking habits and alcoholic beverage consumption (*p* < 0.001) and living with smokers (*p* = 0.003). The enrolled cohort does not seem to need more information about the risks of cigarette smoking (*p* = 0.028). The data analysis and the comparison with the current literature allowed the authors to hypothesise a training model to be adopted within a workplace health promotion programme managed by an occupational physician. This model included targeted training for smoking dissuasion, focusing on sex and gender, cohabitant’s influence, and combined addiction management. Further research will focus on the effectiveness of these proposed models.

## 1. Introduction

Tobacco smoking is one of the most severe public health issues worldwide. Despite numerous efforts to reduce its prevalence, which have enabled an estimated reduction in global prevalence from 25.7% in 2000 to 19.8% in 2015, the tobacco smoking projected prevalence estimated in 2025 will still stand at 17.1%, with significantly higher estimates (24.0%) in Europe. In terms of sex, there is a 3:2 ratio in Europe, compared to 2:1 in the U.S. In other countries, there is an apparent prevalence of tobacco product use in men [1]. Across countries, the mean proportion of current smokers aged 20–54 years in 2019 who initiated smoking by the age of 21 was 76.6% (59.0–97.5). The youngest mean ages at initiation were observed in Europe and the Americas [2].

Smoking will produce a potential 60 million years of life lost within the next 20 years; according to World Health Organization, tobacco smoking is the first leading risk factor causing early death and disability in males [3]. Cigarette smoking is a risk factor for many chronic systemic diseases with inflammatory components such as atherosclerosis, Crohn’s disease, rheumatoid arthritis, psoriasis, ophthalmopathy and non-insulin-dependent diabetes mellitus, in addition to its well-recognised contribution to the pathogenesis of the chronic obstructive pulmonary disease (COPD), hypertension, cardiovascular disease (CVD) and cancer [4,5]. CVD is the leading cause of death in smokers; worldwide, more smokers die from heart disease than those from respiratory disease and all forms of cancer combined [6].

Smoking habits and exposure to occupational risk factors can interact, causing harmful effects on health in both additive and synergistic ways [7]. Indeed, active and passive smoking can lead to severe, often invasive, and sometimes fatal diseases by themselves or through interaction with certain occupational risk factors [8]. In several industrialised countries, a high prevalence of smokers has been found among workers who have low levels of education, who perform low-skilled or low-paying jobs (“blue collar” or “service workers”), and who are also commonly exposed to significant occupational hazards [9]. Smoking has also been linked to a greater risk of accidents at work, fires and explosions: smoking and chewing tobacco, for example, have been associated with a high risk of occupational injuries [10].

In the healthcare setting, according to Nilan K et al., the overall prevalence of tobacco use was 21%, 31% in males and 17% in females, similar to worldwide trends. The country-level comparison suggests that in high-income countries, male healthcare workers tend to have a lower prevalence than males in the general population, while the estimates were similar in females. These data underline that tackling smoking habits among healthcare workers requires urgent action as they are at the front line of tackling tobacco use in their patients [11]. Given the abovementioned issue, the smoking habit is a primary target in workplace health promotion (WHP) programmes, especially in healthcare.

WHP is a coordinated set of activities and strategies at the workplace to encourage the health and safety of all employees. Evidence shows that well-designed, and well-executed WHP programmes can achieve positive health and financial outcomes based on evidence-based principles and through occupational physician (OP) coordination [12].

Effective protection of workers’ health must be based on interventions aimed, on the one hand, at preventing exposure to specific occupational risks and, on the other, at combating unhealthy lifestyles, including smoking tobacco. In addition, healthcare professionals represent a behavioural model to their patients and have enormous potential to play a key role in battling the tobacco epidemic [13].

As a precondition for any intervention in occupational medicine (OM), characterisation of the target population is essential to consolidate knowledge of previous information, even concerning discordant data in the literature. From this perspective, lifestyle habit investigations of an occupational population and its characterisation are fundamental prerequisites for developing a tailored WHP programme in OM. In addition, these studies make it possible to consolidate the experimental model of scientific research and increase knowledge related to public health and OM issues.

The study aimed to investigate the tobacco smoking prevalence, habits and awareness among a cohort of healthcare students from a university hospital in southern Italy and the associations with socio-demographic determinants. A secondary outcome was to estimate the educational needs to receive information on smoking-related risk factors.

## 2. Methods

### 2.1. Study Design, Setting and Participants

The cross-sectional survey was conducted between October 2018 and November 2019 on healthcare students, residents, and PhD students at the University of Campania ‘Luigi Vanvitelli’. The cohort was enrolled among workers undergoing the health surveillance programme required by the University’s occupational medicine section, as required by Italian Legislative Decree 81/08. Participation was voluntary, and subjects signed informed consent and authorised the processing of personal data. The sample was constituted of ‘health students’, including medical and health professional students. The sample size was estimated considering the prevalence of smoking among health professions students set at 50%, using a confidence level of 95% and a margin of error of 5%. This returns a sample size of 385. In addition, this estimated size was adjusted for a non-response rate of 20%, yielding a final target sample population of 481 subjects.

### 2.2. Data Collection, Variables and Outcome

An ad-hoc, anonymous, and self-administered questionnaire was edited for data collection and structured into five sections: socio-demographic and anamnestic characteristics (a); knowledge (b), attitudes (c) and habits regarding cigarette smoking (d); sources and information needs about risk factors related to smoking (e).

The variables selected from the data collected were sex, age, course of study, course year, number of cohabitants and presence of smokers among the cohabitants, alcohol consumption, and comorbidities.

According to the CDC, smoking habits were defined as follows: current smokers, those who smoked 100 cigarettes in their lifetime and who currently smoke cigarettes; never-smokers, those who have never smoked or who have smoked less than 100 cigarettes in their lifetime. Those who have smoked at least 100 cigarettes in their lifetime but who had quit smoking at the time of the survey were defined as former smokers [14].

### 2.3. Statistical Analysis

Statistical analysis was conducted through descriptive and inferential analysis. Descriptive analysis was used to describe the sample’s main characteristics, using average and standard deviation and percentages. Inferential analysis was conducted through a multivariate logistic regression model for the dichotomous outcome “smoking status”, structured as follows: 1 = ‘current smokers’ and 0 = ‘non-smokers’ (including former smokers). Associations were assessed with gender, age, need to receive information on smoking-related risk factors, cohabitation with smokers, and alcohol consumption. Bivariate appropriate tests (*t*-tests, chi-square tests, and Fisher exact test) have been used to assess the associations between independent characteristics and health professions students’ smoking habits. After performing the exploratory bivariate analyses, a multivariate stepwise logistic regression model was performed to assess the independent predictors of the explored patients’ willingness according to the Hosmer and Lemeshow model building strategy. Specifically, only those variables found to be associated at the *p*-value ≤ 0.25 level were introduced into the model [15]. The regression analysis results were expressed as odds ratio (OR) and standard error (ES) with the corresponding 95% confidence intervals. The statistical software used for the analysis was Stata15, and the statistical significance level set of the *p*-value was ≤0.05.

## 3. Results

The questionnaire was sent to 550 subjects, and 549 were finally enrolled (32.8% male and 67.2% female), representing 36% of undergraduate healthcare students who were offered the questionnaire during health surveillance. The average age was 25 (±5.9 SD; range 18–61), and 7% were married. The whole sample attended a health-related course of study; 82% were between their first and third year of the course, 1.5% lived alone, while most cases (89%) had more than one cohabitant, and 86% of the cohabitants were smokers. Concerning alcohol consumption, above 64% reported consuming alcoholic beverages 1 to 3 times per week. Sixty students reported suffering from at least one disease, the most common being thyroid gland pathologies (one-third), while 15 subjects suffered from allergies. A synthesis of sample characteristics is reported in Table 1.

Among smokers (25.3%), 81% started before 18 years old, and 93.3% smoke more than one cigarette a day. Nine subjects only declared the use of e-cigarettes. Overall, slightly more than half of the sample (53.3%) had made more than one attempt to quit smoking; in particular, 62.5% of former smokers revealed that they had made one or more early attempts to quit smoking. Among those who have stopped smoking, 93.7% said they quit smoking ‘to improve their health’. Moreover, 91% of all enrolled smokers would like to quit for the same reason.

**Figure 1:**
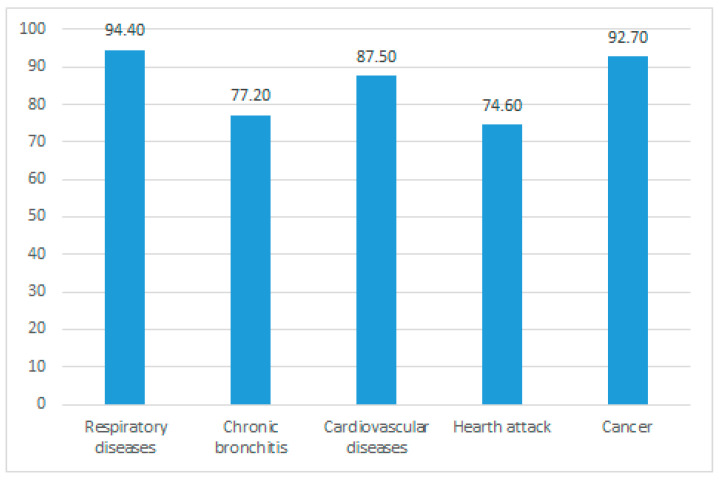
Knowledge about diseases related to smoking habits.

Another investigated issue was the awareness of risk factors related to smoking habits. The overwhelming majority (99.6%) knew that smoking is a risk factor for several diseases (Figure 1). In addition, 97% believed that secondhand smoke is harmful to health, too.

**Figure 2:**
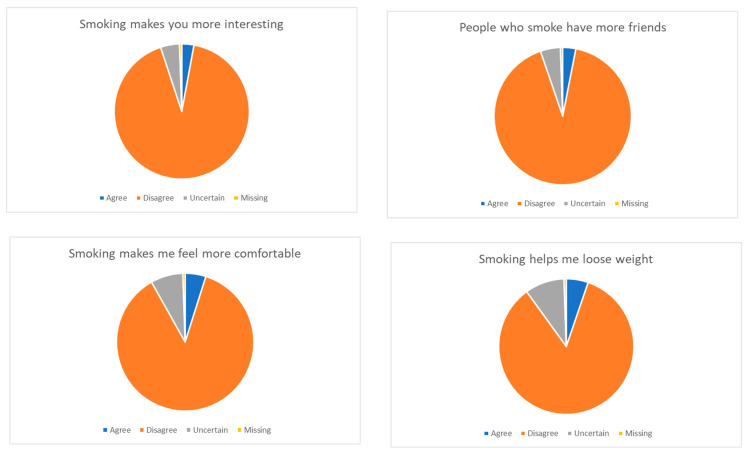
Attitudes and social perceptions toward smoking habits.

Regarding sources of information about smoking-related risk factors, most of the sample (90%) said they had received disclosures from TV/newspapers, the internet, academic sources, and private doctors, but one-third of the whole sample (32.5%) would receive more information. Forty-nine subjects stated they had no knowledge about such risks and did not feel the need to have any. The attitudes and perceptions toward smoking habits are represented in Figure 2.

Most of the sample (87.5%) agreed that “it is right that there should be a ban on smoking in public places”.

The multiple logistic regression model (Table 2) showed that living with people who smoke and consume alcoholic beverages are linked to smoking habits (*p* = 0.003 and *p* < 0.001, respectively), and those who do not want to receive more information about the risks of cigarette smoking (*p* = 0.028) are more frequently smokers.

## 4. Discussion

Total worker health integrates traditional preventive approaches with activities aimed at improving the general well-being of workers. Work represents a crucial determinant of health since many work-related factors (such as wages, working hours, relationships with colleagues and superiors, access to holidays, and the relationship between work and private life) have an important impact on the well-being of workers, their families and their communities [16]. Therefore, the workplace represents the ideal setting for health promotion interventions, such as smoking dissuasion. Smoking bans should be mandatory in occupational settings. However, it is also essential to protect workers from secondhand smoke, provide information on the risks of smoking, and offer smokers the opportunity to fight addiction with these preventive measures, as part of the WHP’s basic assumptions.

This research aimed to analyse the prevalence of smoking habits among health profession students and the association with socio-demographic determinants. In addition, personal attitudes were also examined to identify insights for planning promotion activities among health professionals.

Several insights, concerning the main topic of interest, arose from the comparison with related literature work (Table 3).

The prevalence of current smokers in our sample was 25.3%, in line with Cena H. et al. [17] and Gallè F. et al. [18], and which stands between two other available data from Italy, with a prevalence ranging from 20.9% [19] to above 31.3% [20]. This last data came from a European survey that reported a higher prevalence among medical students than the general population, with the highest prevalence in Italy.

In our sample, about 34.2% smoked at least once in their lifetime; this result disagrees with La Torre et al. [19], who report 73% of these subjects. This difference in the data could be attributed to the different study design or data collection periods (2012 vs. 2019) and thus to different eras in which more recent, persuasive smoking cessation campaigns may have been conducted.

Regarding sex distribution, the sample randomly recruited participants at a male-to-female ratio of almost 1:2. These data indeed reflect the current sex distribution in medical degree programmes in Italy, as shown in an Italian survey published last March 2022, where it appears that the female sex is more prevalent under 50 years old [21]. Our sample showed that among all the smokers (former and current), 37.7% were male while 62.8% were female. However, after adopting a logistic regression model, our sample results showed no gender differences among the students involved (Table 2). Although not found to be statistically significant, the prevalence of smoking by women appears to be in partial contrast to the global data on smoking habits [1]. The Italian data from a cohort study among medical residents in public health showed a similar trend [19]. Our study examined sex differences exclusively, whereas other authors analysed differences in smoking habits between gender identity and sexual orientation, showing no significant results [22]. These aspects are of current interest but, at the same time, may present some ethical problems in study designs and survey settings.

Our analysis also showed that the prevalence of smoking among students in the first three years (26%) and those in later years (25%) is similar and overlaps with the overall incidence in our sample. No clear indication emerges from these data as to a specific time to begin specific training.

Smoking habit is strongly influenced by family members and cohabitants [23]. Our logistic regression model confirmed this finding with a statistical significance for the association between cigarette smoking and cohabitation with smokers, according to several studies that emphasise the harmful impact of living with smokers on the influence of a cigarette smoking habit, particularly on smoking cessation behaviours [24,25].

According to the literature, we also showed an association with people who smoke and consume alcoholic beverages [26,27]. These combined habits are often related to work presenteeism, perhaps as a misguided coping strategy to deal with work stress. This association could indirectly indicate employee job stress, so companies should build appropriate strategies and programmes to help reduce these behaviours [28]. Other determinants, such as dietary habits, were investigated by different research groups. According to Bravini et al., being a smoker is associated with worse nutritional habits [29]. Our study did not explore these associations, representing a field of exploration for future investigations.

An additional item to be considered is the electronic cigarette smoking habits, developed over the past few years and introduced into the Italian market in 2010 [30]. In our current smokers’ sample, 12.7% declared themselves to be e-smokers, with only three subjects said to be dual users. In Europe and Italy, few data on e-cigarette prevalence among medical students are available. Kinnunen et al. reported an average prevalence of 30% of e-cigarette smokers among European adolescents [31]. It should be mentioned that the introduction of new tobacco- and nicotine-based products (from e-cigarettes to new-generation non-smoking products) on the market has opened new scenarios regarding prevention strategies. The spread of such products represents a cause for interest and public health concern. Indeed, the scientific literature is not exhaustive, neither about the long-term health consequences of using such products and their lower level of harmfulness nor the effects of passive exposure on their emissions.

This study was conducted in a population that should be considered an elective target for health promotion programmes for two reasons. On the one hand, the cohort is represented by young adults who have approached smoking as a kind of social integration model. Smokers reported better social integration and massive influence from smoking friends, to such an extent that better social integration and intimacy with friends are more important than the reduction in subjective and emotional health. Social smokers’ determinants may fail to recognise the health risks associated with tobacco use, so intervening with this group is a challenge [32]. In health campaigns, the best communication approach is to focus media interventions on younger segments of the student population to dismantle false stereotypes, as reported by Mannocci et al. [33].

On the other hand, medical students represent a population that will exercise a health protection role in public and occupational settings. Therefore, they should be more sensitised about health promotion issues. Medical students should be aware that healthcare professionals play a key role as models for the general population and that they may receive specific training in counselling patients on smoking dissuasion. A high prevalence of smokers among these students should limit the implementation of awareness programmes against cigarette smoking because they would not turn out to be suitable role models. Smoking health professionals constitute a barrier in smoking cessation conversations, and convincing the patient may become harder.

The literature research regarding health promotion strategies to reduce tobacco demand in adolescents, youth and young adults showed discordant experiences concerning the effectiveness of these strategies [34]. In a meta-analysis performed in 2019, the authors showed a success rate in the application of smoking cessation interventions of 21%. Behavioural, pharmacological or policy intervention could obtain positive results in quitting smoking among healthcare workers, better if combination approaches were introduced [35].

A still-high prevalence of smokers among our target population (about 25%) could be explained by ineffective health promotion programmes during academic courses and increased pressures/academic stress during the course. In our opinion, health professional students may need training about nicotine addiction and tobacco cessation in their core university curriculum to help them stop smoking and influence patients’ smoking behaviour. As reported by D’Egidio et al., a high percentage of healthcare students agreed with the ban on smoking in public and enclosed places and confirmed the necessity of receiving specific training on smoking cessation techniques, given that only 24% received specific training during the academic course [36].

The results of La Torre et al. performed an intervention that showed an effect in significantly reducing the number of smokers after specific training, supported this statement [37].

Within training programmes, a critical aspect is the limited knowledge of the emerging science of e-cigarettes and heat-not-burn products. Therefore, additional training for undergraduate and graduate medical students is strongly encouraged because such products are often promoted as smoking cessation devices that are less harmful than conventional cigarettes. Despite all, there is evidence that they may cause cardiovascular damage and lung inflammation [38].

**Table 3:** Related work summary.

Topic	References
Smoking habit frequency	[17,18,19,20]
Determinants	
– sex and gender	[19,21,22]
– cohabitants	[23,24,25]
– combined alcohol consumption and nutritional habits	[26,27,28,29]
Intervention programmes	[34,35,36,37,38]

According to Warren et al. [39], health institutions have a moral duty to help their students quit smoking by providing encouragement and information to students considering stopping and helping those motivated to quit. Limited and inconsistent levels of tobacco training are currently being provided to healthcare students; minimal education about smoking-related issues in public health programmes is reported [19].

In our sample, about 90% declared they had received information about the risk factors related to smoking through multiple sources. This finding probably justifies the association between smoking habits and reluctance to receive more information about the risks of cigarette smoking, as found in our research. Regarding the information sources, 4.7% of participants reported receiving details exclusively from university courses, while more generally, about 58% were from different sources, including academic ones.

**Figure 3:**
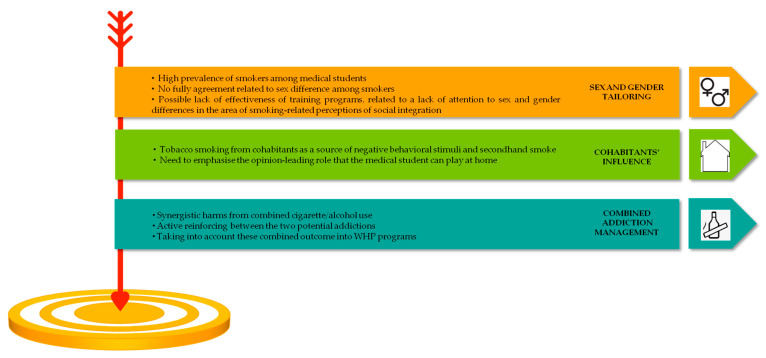
Insight for workplace health promotion programmes related to smoking dissuasion. A key point of the intervention should be the awareness improvement of the role model for patients in the target population.

The results of our research offer a variety of valuable insights into the creation of WHP programmes for smoking deterrence. Figure 3 shows a framework for such programmes.

This information can be conveyed through two main channels. The first is undoubtedly the educational programmes provided by the course of study. E-learning courses may serve as a theoretical basis that could ideally be complemented with additional training in clinical intervention [38]. Since no significant differences in smoking habits were found between the different years of the course, it must be assumed that these topics can be introduced at various stages of the degree course, also concerning the different stages of acquiring awareness of one’s role within the healthcare system. This stratification can also affect the different modes of training delivery, moving from formal didactics to more specific tools such as focus groups, not ignoring more modern communication channels (such as social or websites), which can be more persuasive to millennials. Dedicated efforts to prevent students from starting smoking, help current smokers quit, and instil a sense of responsibility as a role model in future patients should be incorporated into their curriculum as early as the first year.

In this context, the OP can contribute to WHP by having several opportunities and possibilities to encourage smoking cessation. First, the OP has a key role in understanding the interactions between smoking and occupational risk factors, being the only figure in the workplace who can produce targeted and qualified information on these risk interactions. In addition, the OP should implement training on the significant benefits of smoking cessation and the reduction of smokers’ prevalence among employees by considering smoking in the analysis of the psychological and physical well-being of smoking and non-smoking workers [40], including through brief counselling of tobacco users during health surveillance screenings, aimed at the opportunity to quit smoking and initiate cessation. Such interventions are effective both when provided in the workplace and other settings, complementing and personalising the initiatives and projects that public health professionals lead.

This study has some limitations. First, while the survey completion rate was high, the participation rate was about 36%, and no detailed information about non-responders was collected. In addition, the study used a self-administered questionnaire without free comments from the interviewees. Lastly, the cross-sectional design and data self-reporting could have led to weak information estimations.

Despite these limitations, the present research has several strengths. The sample size was large, with various and homogeneous representations of study courses (such as nurses, physiotherapists, orthoptists, midwives, laboratory and radiology technicians, dental hygienists, pharmacists, and biologists). From a methodological point of view, the enrollment was performed according to a non-arbitrary definition of smoking status but following the CDC’s standard definition, allowing for comparison with previous and future research.

Furthermore, the prevalence data of smoking habits align with other Italian statistics, which were comprehensively examined in the discussion.

The results of this study have relevant local and general implications. Based on the investigation, it was possible to design a study protocol as part of an occupational health promotion programme managed by the occupational physician. The study suffered a start-up slowdown due to the onset of the pandemic but is imminent and will allow the evaluation of the effectiveness of a smoking cessation intervention programme focusing on sex and gender, cohabitant influence and combined addiction management. A companion paper will be produced later, on the effectiveness of the proposed training model.

More generally, to complete the picture of young adults’ lifestyles in this context, further surveys based on different collection methods and including people who attend more than just universities are needed.

## 5. Conclusions

The survey results showed that the smoking habit is frequent among health students. Implementing active information on health promotion and smoking-related risk factors is essential by introducing specific training within university courses (aimed at increasing health professionals’ awareness of health promotion tools/smoking cessation courses as secondary prevention).

The occupational physician, in synergy with the employer, can be an active part of this process by implementing preventive interventions in the workplace through an equity-oriented approach, which helps build targeted WHP programmes in specific work settings. Further investigation, possibly with a high-quality methodological approach and field-based, additional to public health programmes, could redefine the true prevalence of the phenomenon and its associated determinants. Studies on workers’ habits and lifestyles can be the starting point for drafting a specific health promotion programme aimed at workers and targeted to the peculiar needs related to workers’ habits.

## Figures and Tables

**Table 1:** Main socio-demographic and anamnestic characteristics of the sample.

	*n*	%
**Age**		
<25 yo	296	53.9
25–34 yo	216	39.3
≥35 yo	37	6.8
**Sex**		
M	180	32.8
F	369	67.2
**Study Course**		
Medical Students	88	16.0
Health-Professional Students	273	49.7
Residents	167	30.4
Others	21	3.9
**Course Year**		
1st to 3rd year	446	81.2
>3rd year	100	18.2
N/A	3	0.6
**Smoking Habits**		
Y	139	25.3
N	361	65.7
Former	49	9.0
**No. of smokers cohabitants**		
0	318	57.9
1	146	26.6
>1	76	13.8
None cohabitants	8	1.5
N/A	1	0.2
**Alcohol consumption**		
Rarely (≤once per week)	96	17.5
Sometimes (1–3 times per week)	257	46.8
Often (≥4 times per week)	6	1.1
Every day	10	1.8
Never	180	32.8
**Diseases**		
At least one	60	10.9
No pathologies	489	89.1

**Table 2:** Multiple logistic regression model.

Variable: Tobacco Smoking	OR	ES	IC 95%	*p*
Female gender	0.73	0.15	0.49–1.49	0.128
Age 25–34 yo	0.71	0.15	0.47–1.07	0.106
Age ≥ 35 yo	0.57	0.27	0.23–1.42	0.229
Need to receive information on smoking-related risk factors	0.62	0.13	0.40–0.95	**0.028**
Cohabitation with smokers	2.19	0.57	1.31–4.02	**0.003**
Alcohol consumption	2.56	0.59	1.63–4.02	**0.000**

Log likelihood = −316.6, χ^2^ = 45.8 (5 df). Statistically significant *p* values are highlighted in bold.

## Data Availability

The data presented in this study are available on reasonable request from the supervisor author.
